# Screening and referral is not enough: a qualitative exploration of barriers to access and uptake of mental health services in patients with cardiovascular diseases

**DOI:** 10.1186/s12913-020-06030-7

**Published:** 2021-01-08

**Authors:** C. M. Collopy, S. M. Cosh, P. J. Tully

**Affiliations:** 1 School of Psychology, University of New England, Armidale, NSW 2351 Australia; 2 Freemasons Centre for Men’s Health, School of Medicine, University of Adelaide, Adelaide, SA Australia

**Keywords:** Service use, Depression, Anxiety, Depression screening, Help seeking, Mental wellbeing, Mental health treatment, Coronary heart disease (CHD), Mental health services, Thematic analysis

## Abstract

**Background:**

Cardiovascular diseases (CVD) are commonly comorbid with mental health disorders, portending poorer cardiac prognosis. Despite the high prevalence of depression and anxiety, and guidelines recommending routine depression screening and referral, uptake of mental healthcare in CVD populations remains low. Reasons for the underutilisation of mental health and psychological services for this population remain largely unknown.

**Methods:**

Thirteen CVD patients with clinically significant psychological symptoms (depression, anxiety and/or stress) participated in one-on-one in-depth semi-structured interviews. Data were analysed using inductive thematic analysis.

**Results:**

Barriers to uptake included the timing of referral and screening, with patients reporting a need for longer term follow-up. A lack of information provision and understanding around mental health and services, especially following cardiac-events were further barriers. A reluctance to report mental health or engage in services was also identified, with patients indicating a preference for informal peer support networks. A range of practical barriers such as mobility, transport and cost were also reported.

**Conclusions:**

Longer term follow-up and routine mental health assessment may be beneficial to facilitate use of mental health services. Upskilling of practitioners around mental health may be a further avenue to promote information provision and enhance service use. Further focus on enhancing informal peer support may be a valuable initial approach for the CVD population. The implications for improving services and enhancing service use are discussed.

## Background

Cardiovascular diseases (CVD) are the leading cause of disability, disease and mortality burden globally [1]. Comorbid depression and anxiety disorders are common, with estimates suggesting that 15–20% of CVD patients have depression [2] and 15% have an anxiety disorder [3]. Comorbid mental health disorders pose a two- to three-fold increased risk of morbidity and mortality within six months of cardiac event [4]. Mental health symptoms also impinge on a person’s ability to participate in secondary prevention such as medication adherence and cardiac rehabilitation, and increase the likelihood of unhealthy lifestyle behaviours [5–7].

The deleterious impact of mental health disorders on quality of life and cardiac outcomes is underscored by the recommendation for routine depression screening in cardiology guidelines around the world (e.g., [8–13]). Despite recommendations and implementation in many countries, routine screening has not resulted in changes to quality-adjusted life-years or depression-free days [14]. This may be due to low uptake of treatment, with few patients pursuing mental healthcare after routine screening and referral [15, 16]. For example, in an English cluster-randomised trial, 33% of CVD patients referred to collaborative depression care did not attend any depression treatment [17]. Similarly, only 36% of patients referred for consultant-psychiatry post-acute coronary syndrome accepted the referral [18]. Further studies in the US and Australia have found that as few as 2% accessed mental health services following depression screen and referral [19, 20].

When accessed, mental health interventions appear acceptable [21, 22], yet specifically why this population tends not to utilise dedicated mental health services even after a positive depression screen and referral remains unclear. The broader literature highlights a number of barriers to mental health help seeking, such as hegemonic masculinity, fear of disclosure, stigma, and concerns around judgement from others [23, 24], as well as financial barriers, and symptom severity [25]. The extent to which these barriers influence help seeking for mental healthcare in CVD populations is less certain. Further, the low uptake of mental healthcare in CVD populations, despite dedicated screening and referral, suggests there may be further barriers specific to this population. Yet these barriers remain largely unknown and unexamined. A study of coronary heart disease (CHD) patients’ perceptions of mental health indicated a reluctance to use anti-depressant medication or to discuss their mental health with their general practitioner [22]; however, reasons for this reluctance were not explored in depth. Thus, to date, the barriers to mental health help seeking and service use within CVD populations has not been extensively examined.

Given the low uptake and utilisation of mental health services following routine depression screening, identification of barriers to help seeking is imperative; especially given that poor mental health portends poorer CVD outcomes and increases mortality risk. Accordingly, this study aims to better elucidate the barriers to accessing mental healthcare specific to CVD patients with a clinically significant psychological condition.

## Method

### Design

This study is exploratory given the limited extant knowledge regarding reasons for mental health service underutilisation in this population, thus this study employed a qualitative design, using individual in depth interviews. This study was undertaken in the mid-North region of New South Wales, Australia; a rural region with a population catchment of more than 300,000 persons. This region is representative of non-metropolitan areas in NSW and eligible patients were invited from a range of health clinics in this region to participate in the study.

### Participants

Thirteen participants completed interviews in late 2018-early 2019. To be eligible, participants had to have a) diagnosis of any CVD, b) clinically significant symptoms that would necessitate treatment in at least one of depression, anxiety, or stress; as assessed by a score above clinical cut-off on the Depression Anxiety and Stress Scale–21 (DASS-21 [26];), and c) received a mental health referral. Of the participants, six were male and seven were female, and presented with a variety of CVDs (see Table 1). Ages ranged from 34 to 80 (M = 62; SD = 12.09) years. Data saturation was deemed to have occurred after nine interviews, with a further four completed to ensure that no novel themes or ideas emerged [27]. No further interviews were deemed necessary due to saturation.

**Table 1:** Participant profile characteristics and demographics

	Pseudonym	Age (yrs)	Cardiovascular history
1	Mary	80s	CHD with PCI
2	Maggie	60s	CHD
3	Margaret	60s	AF
4	Adam	60s	MI with PCI
5	Marjorie	60s	MI with PCI
6	Melanie	50s	MI with PCI
7	Michael	70s	MI with CABG, pacemaker insertion
8	Sam	60s	MI with CABG
9	Joe	60s	CHD with CABG
10	Dan	40s	CHD with PCI, SCA
11	Melissa	30s	Congenital heart disease, cardiomyopathy
12	Jim	60s	MI with CABG
13	Mena	70s	AF

*AF* atrial fibrillation, *CHD* coronary heart disease, *CABG* coronary artery bypass graft, *MI* myocardial infarction, *PCI* percutaneous coronary intervention, *SCA* sudden cardiac arrest

### Procedure

Following approval from the University of New England’s Human Research Ethics committee, a flyer (which advertised the study as aimed at better understanding the impact of CVDs on quality of life and wellbeing and access to care for wellbeing) was placed on noticeboards in medical practices, hospitals, and community health services in the mid-North coast region of NSW, Australia. Interested volunteers contacted the researcher through email or phone to determine eligibility (including completion of the DASS-21) and to schedule an interview once eligibility had been ascertained. Interviews took place at a location of the participant’s choosing, such as their own homes or private rooms in local libraries and community centres. Prior to interviews, participants provided written informed consent and completed a brief demographic questionnaire. Interviews were audio recorded and recordings were transcribed verbatim. Pseudonyms were used in all transcripts to ensure anonymity and any potentially identifying information was removed from all transcripts.

### Measures

#### Demographics

Participants completed a short demographic questionnaire to obtain age, gender, CVD, medical conditions, and history of mental health diagnoses.

#### Psychological symptoms

The DASS-21 is a well-validated 21-item measure of psychological distress, assessing three conditions: depression, anxiety and stress [26]. Participants are asked to specify the frequency, on a 4-point Likert-scale (never to almost always), with which they have experienced symptoms. There are seven items assessing each condition (e.g. “I couldn’t seem to experience any positive feeling at all”, “I feel scared without any good reason”, and “I found it hard to wind down”). The DASS-21 has good psychometric properties (Lovibond & Lovibond, 1995). There are well established clinical cut-offs for each condition (depression, anxiety and stress), with high sensitivity and specificity for predicting diagnosis (e.g., [26, 28]). Moreover, the DASS-21 has been commonly used with cardiac populations (e.g., [29, 30]).

### Interviews

Participants took part in semi-structured interviews with a provisional psychologist (final year of clinical Masters training) that were approximately 1 h in duration. A list of questions were developed in consultation with the co-authors (with relevant clinical and research experience as psychologists and researchers in health and cardiac settings), as well as the extant literature. The questions were designed to provide participants with an opportunity to convey their experience. Interviews commenced by asking participants to describe their experience of living with CVD. Participants were then asked additional questions regarding their mental health and reasons for seeking or not seeking mental healthcare (see Supplementary Material for interview schedule).

### Data analysis

Data were analysed using inductive thematic analysis [31], rooted in a realist epistemology. That is, participants’ responses are understood to convey their intended meaning and accurately reflect their own experiences, emotions and thoughts. Phase one of the data analysis involved reading and re-reading the dataset to develop familiarity of the data. Phase two involved generating codes that identified important semantic and latent elements related to the research question. In phase three, coded data extracts were collated together and scrutinised to identify significant recurring patterns of responses across the dataset relating to mental health help seeking. Initial themes were then determined based on the prevalence of recurring responses across the dataset as well as the salience of themes for answering the research question [32]. Each initial theme was re-examined to determine coherence and consistency. From this process, three overarching global themes that encompassed a number of sub-themes were identified. During the final phase, sub-themes were refined, to ensure the theme was representative of the global theme and accurately depicted the meanings evident within the whole dataset [32]. Following this phase the second author reviewed identified themes to determine if they adequately represented the coded extracts of the entire dataset and determined whether each thematic label encapsulated the data they represented, in order to ensure analytic rigour (for a detailed discussion on rigour in qualitative research see [33]).

## Results

Of the sample, 8 participants met clinical cut-off for depression, 10 for anxiety, and 8 for stress (with multiple participants in the clinical range for more than one condition), thus all participants reported symptom levels that would necessitate treatment. Three interviewees also disclosed suicidal ideation and suicide attempts since the onset of their CVD. Despite elevated symptoms and receiving mental health referrals, only 2 of 13 (15%) interviewees had sought mental healthcare. During the interviews, participants were asked to elaborate on why they had not sought help. Three key themes were identified – information provision, openness to mental health and healthcare, and practical barriers - underpinning reduced mental health help seeking. Each of the themes also contained several subthemes, further elucidating the underutilisation of mental health services.

### Information provision

When asked to outline reasons for not accessing mental health services, participants repeatedly oriented to a lack of information provision with regards to mental health and its relationship with CVD.

#### Mental health-CVD link

Almost all interviewees reported that they had initially been unaware of the well-established relationship between CVDs and mental health. In particular, most stated that they had not been made aware of the high rates of depression and anxiety following cardiac events. Thus, for many, they were unprepared for resultant poor mental health and were less aware of what they were experiencing or how to manage it. A lack of information provision regarding the mental health-CVD link, resulting in limited understanding of participants’ own experiences, is illustrated in the extract below.

*You don’t know what is normal, how you’re supposed to feel. I mean how are we supposed to know how we are going to feel in any given situation* (Melanie, MI with PCI)

In the few instances where information regarding the CVD-mental health relationship was provided, interviewees reported that this information helped them to prepare for and understand their experiences. Moreover, upon receiving information, these participants described a sense of relief that their subsequent depression/anxiety were “*normal”,* and that there was a “*reason for feeling blue”.* The below extract highlights the relief reported upon being provided with mental health-CVD information.

*The mental side of it, it was so extreme … I didn’t realise, you know, till someone pointed it out. But you know one of the nurses actually in the cardio program said, “You’re one of those that gets affected by this.” I said, “What do you mean?” She said there’s something like 30%, I think that was the figure. Who get really affected by open heart surgery, they suffer. I thought thank God you know. I said, “thanks for that,” I said, “I thought I was going mad”. She said, “No it’s a real thing”. She said, “there’s marriage break ups, there’s depression, anxiety.” I thought, “Oh that’s a relief”* (Sam, MI with CABG)

#### Understanding of mental health

While a number of participants reported receiving brochures or some information regarding mental health, none reported having a conversation that helped them to understand what mental health concerns ‘looked like’. That is, interviewees were typically unaware of how to identify symptoms of poor mental health. Consequently, participants described being unaware of how their experiences fitted within mental health. This lack of understanding also left them unsure of how to make sense of their emotional experiences post-CVD onset; as is illustrated below.

*Michael: I needed information badly but nobody talked to me.*

*Interviewer: And that information was about what’s going on in my body, what’s going on in my head?*

*Michael: Yeah. Why do I feel this way. You know, why am I… Instead of thinking positive things, thinking on the negative side all the time and I became very very negative.*

*(Michael, MI with CABG, pacemaker insertion)*

*Adam: Ahh, just my GP told me that I’d, that I should be aware that you might suffer depression*

*Interviewer: Did they give you an idea of what those symptoms might be like?*

*Adam: Nah*

*(Adam, MI with PCI)*

### Openness to mental health and mental health services

#### “I’m not depressed”: lack of acknowledgement of mental health

Despite having clinically significant symptoms consistent with a psychiatric disorder (assessed by the DASS-21), interviewees did not see themselves has having a mental health condition. All but three interviewees resisted mental health nomenclature, including those interviewees who had reported suicide attempts. Rather, interviewees used language such as ‘teary’, ‘feeling vulnerable’, ‘frustrated’ or ‘adjusting’ to describe themselves, and actively disavowed diagnostic categories; also potentially highlighting the lack of mental health awareness and knowledge. Examples of language and disavowal of diagnostic terms are evidenced in the extracts below:

*I think sometimes, I think, I think there have been occasions lately where I’ve really felt down and I’ve really felt teary* (Mena, AF)

*I’m not depressed. I mean I can go to a very dark place in my mind, but I’m not like that all the time … and I try to be fairly upbeat with people no matter how I’m feeling* (Majorie, MI)

#### “It’s not for me”: lack of engagement with mental health services

Relatedly, interviewees most commonly stated that they would not be interested in seeking support for their mental health and did not action a referral. This was, in part, due to not seeing themselves as having a mental health condition and thus not viewing support as necessary. Additionally, for the majority of interviewees, understanding and acceptability of psychology and mental health services was low, with many reporting that it was not something they would be interested in (that they were “*not ahh into that sort of thing*” or “*it’s just not for me”)*, such as is highlighted below.

*If there were notes being taken and all the rest of it people wouldn’t respond. I know I wouldn’t personally. I’d think, ‘oh well they’re just sticking their bibs in where they’re not bloody wanted’… they do too much deep probing and I’m not into that sort of thing* (Michael, MI with CABG, pacemaker insertion)

In addition to a disinterest in seeking support, the majority of interviewees also indicated a belief that mental health services would not be helpful for their circumstance. Notably, even those who reported suicidal ideation also described mental health services as not needed, such as is highlighted below:

*Dan: At this stage I don’t think anything is going to change my outlook. I’m just set in my ways.*

*Interviewer: Okay. So you don’t think that going to see a mental health professional would help you like that?*

*Dan: No, I don’t. I’ll be honest with you. I think I’m beyond help. (laugh/cry)*

*(Dan, CHD with PCI, SCA)*

*I don’t really think I need to talk to anyone. I mean I don’t really know it’s ah you know sometimes I’ll think “Oh pull yourself together”* (Marjorie, MI with PCI)

Also evident in the extract from Marjorie, was a sense that patients should be able to manage their emotional wellbeing themselves. Typically interviewees presented themselves in stoic terms; depicting themselves as able to (or that they *should* be able to) cope and manage with their CVD over time. Interviewees frequently reported a preference to “*just sort of get on with my life*” (Marjorie) or “*just put it aside and get on with it*” (Melanie). Participants did not foresee a role of mental health professionals to support or aid learning about how to cope with their CVD. Additional examples of stoicism can be seen in the extracts below.

*Um one adjusts, I’ve adjusted all my life… Everybody in my age group are losing partners and everything – they’re adjusting all the time. And they’re managing all the time. Um its, its, if you don’t do it nobody else can do it for you so* (Mary, CHD with PCI)

*I um I just got on with it. I eventually got on with it. And I think I just buried all those feelings…I guess it was the way I’ve always coped with things I just tried to forget about it* (Melissa, Congenital heart disease, cardiomyopathy)

Thus, evident throughout the interviews was that the overall buy-in to the value of mental health services was low throughout the sample interviewed, resulting in low uptake of mental health referrals.

#### Preference for informal support

Although interest in accessing formal mental health support was low, almost all participants reported that they would have preferred attending dedicated peer support groups with other individuals “*going through the same thing*” rather than formal mental health services. Most participants attended cardiac rehabilitation and other cardiac-related groups (e.g., walking groups) and described their interactions in these groups as valuable, furthering their desire and preference for dedicated mental health support groups. This was especially evident from the younger participants, who reported a lack of same aged peers when attending cardiac rehabilitation or other cardiac-related programs and groups. A less formal, peer-based support appeared to be more acceptable for the interviewees than seeing a psychologist, at least as an initial step. The preference for peer networks is highlighted in the extracts below.

*Have groups of um heart patients that can get together and talk about the mental side of it* (Sam, MI with CABG)

*So like support groups like a um a group that you could go to where you could go to talk about your problems.* (Melanie, MI with PCI)

*I’m sure that if ah I was told ‘Oh look there’s half a dozen people under the age forty ah who have had cardiac event surgery, you know why don’t yous [sic] get together and have a chat about how yous [sic] feel’, … that would be beneficial* (Melissa, Congenital heart disease, cardiomyopathy)

### Practical barriers

When explaining what had prevented them from accessing mental health support, ten interviewees also cited a range of practical barriers, including the timing of referral, mobility/transport limitations, and costs.

#### Timing

A common subtheme was reference to the timing of referral or provision of information regarding mental health. The majority of participants reported that the information/referral they received was “*too soon*” in their CVD journey. For many, recovering from hospital admissions and surgeries, as well as adjusting to their physical ill-health took time and left them under-resourced to process mental health information and referrals. Consequently, mental health information and referrals were not followed up or actioned. The below example, in response to being asked why he had not pursued his referral, highlights the timing of information as a barrier.

*It was nearly three weeks before I got out of bed. Before I could even think of it*. (Michael, MI with CABG, pacemaker insertion)

Many participants also reported being overwhelmed when in hospital and shortly after returning home by the experience, as well as the large volume of information and brochures that they were receiving, such as for nutrition, exercise, and other aspects of CVD management. For many, the volume of information, in addition to physical ill-health, resulted in them not reading or absorbing the information. Consequently, mental health information or referrals were not followed up or actioned. An example is provided below:

*You know, [everyone has] got so much on their minds they might not physically be able to make the phone call. And you get given so many like pamphlets regarding like the physical exercise and what you should and shouldn’t eat and so you might not even read it* (Melissa, Congenital heart disease, cardiomyopathy)

Further, many participants reported initially feeling as though they were coping with their CVD, typically reporting higher levels of social and familial support in the period shortly after CVD-onset. Rather, it was several months post-CVD diagnosis before many participants began to feel as though they were not adjusting or coping. Interviewees stated that sustained changes in lifestyle took time to become aware of, with changes to mobility and functioning not always fully apparent until some months later. Most interviewees indicated that they would have liked mental health information and referral to be provided to them later post-CVD onset, indicating that they would have been more receptive to referrals and information at a later stage. These interviewees also reported that mental health assessments over the longer term would have been beneficial and may have prompted them to seek help, as is evidenced in the below examples. The first comes from the response to a question about what the interviewee wished she had received in terms of mental health follow-up. The second comes in response to the question of what timing the interviewee would have preferred regarding mental health follow-up and referral.

*And not just the once with, with the follow up visits because people process things and something they are not aware of they might need at stage one they might need at stage 2. So if it’s brought up with every follow-up visit. “Do you now feel …* (Maggie, CHD)

*Oh probably like I say you know post-surgery you know within a month, two months as you recover and probably you know 3 years, 5 years, 10 years all of those.* (Joe, CHD with CABG)

Interviewees also reported that as the disease progressed, their functional ability was further impacted, compounding reductions in quality of life. Several interviewees reported that they needed help later in the CVD progression when their independence and ability had deteriorated, as is highlighted in the extracts below, yet routine screening is not common at these later stages.

*Yeah a couple of years after the heart attack, I was visiting me GP and um the depression was really starting to kick in then* (Joe, CHD with CABG)

*Michael: I needed somebody to talk to, I got a friend I rely on. He actually saved my life ‘cause I attempted suicide twice …*

*Interviewer: How long after you had the operation down in [hospital] did you start to feel like that?*

*Michael: That was probably 2 nearly 3 years*

*(Michael, MI with CABG, pacemaker insertion)*

#### Access barriers

Seven participants cited costs associated with mental healthcare as prohibitive (at the time of interviews, 6–10 psychologist sessions per year could be accessed free of charge in Australia, although awareness of this was low in the sample). Further, several interviewees referenced their inability to stay in employment, which further compounded financial barriers to services. For some of these participants, mental healthcare was not seen as a priority given limited funds. Examples of cost as a barrier are evident below, with the second example also illustrating the additional burden of unemployment.

*But it was um, something like I would go and I would be there for maybe 30 minutes and it was a cost of about $195, for which when I walked out, I could go to the health benefit place in [city] and I would get back, say the $100 but it still, it still cost me $95 to do that…Plus petrol to get over to [town] and I didn’t think it was worth it financially* (Mena, 74, AF)

*Unfortunately I’m in a state of unemployment at the moment and ah I just don’t have spare money. I I I would love to go and see somebody I think* (Dan, CHD with PCI, SCA)

Several interviewees also cited poor mobility and being less physically capable due to their CVD as barriers to help seeking, which also increased difficulty with transport to attend sessions. In addition, many interviewees reported having a high number of medical appointments for their physical condition(s), with attending mental health appointments viewed as burdensome due to challenges in attending multiple appointments, as is illustrated below.

Y*our ability to travel is inhibited … getting on and off public transport in the city is pretty daunting if you’re on your own in the crowds too, but the physical things are getting to it um because my system is now blocking up um I’ve got a leg swelling and getting to and from is virtually impossible at times* (Maggie, CHD).

*That’s the thing I’ve found like since the heart operation so many thing have happened. Like I’ve got cellulitis, I’ve got neutropenia my white blood cells that could be my tiredness, I’m borderline ah bad neutropenia where my white, bone marrow does not produce neutropene’s anymore to fight infection in my body and that’s only since the operation so somethings happened … I’ve got cellulitis I’ve got vertigo, all these things that just it’s just one thing after another you know.* (Sam, MI with CABG)

## Discussion

This study examined the barriers to mental health help seeking in a population of patients with CVD and comorbid psychological disorders. Within the sample interviewed, uptake of mental health services was low (15%). A range of barriers to uptake that were specific to the CVD population were identified, beyond barriers already identified for help seeking in general mental health populations (e.g., Doblyte & Jimenez-Mejias, 2017). Specific barriers included a lack of information provision, especially regarding mental health post-CVD, and low openness to mental health services. Further, the timing of screening, referral and provision of mental health information was described as suboptimal, suggesting that current screening practices proximal to an index CVD admission might not be best placed to address mental health burden in this population. A range of additional practical barriers including access issues and cost were also noted, consistent with barriers to participating in cardiac rehabilitation (e.g., [34]) and CVD prevention programs [35]. Additionally, participants reported a preference for informal peer support networks rather than traditional mental health services. The study’s findings highlight key areas through which health services related to mental health screening and management in cardiac populations might be able to be improved in order to address the low uptake of mental healthcare.

A lack of information provision regarding emotional experiences post CVD-event was repeatedly reported, echoing previous findings [36, 37]. The lack of information was described as impeding understanding of mental health concerns and emotional experiences post-CVD diagnosis. Likewise, prior research has also identified a lack of understanding of depression or connection between physical ill-health and experiences of depression in a CHD sample [22]. Such a lack of understanding of depression and anxiety following CVD onset posed a notable barrier to help seeking in our sample. Concomitantly, where participants reported that information was provided, this was described as having normalised the experience and prompted accessing mental health services. Although cardiac patients report a preference to receive information either verbally or in pamphlet-form post discharge, this rarely occurs [36]. Notably, where patients received targeted feedback regarding depression in addition to screening, patients were more likely to seek further depression information and had reduced depression severity at 6 months compared with patients who received screening only [38]. Thus, an enhanced focus on information provision around common co-morbidity of mental health and CVDs in health service provision might assist in normalising such experiences and increasing mental health literacy, which may facilitate uptake of mental health referral. Lack of information provision may be due partly to practitioners lacking knowledge of mental health disorders, as well as lacking confidence discussing mental health with patients [37]. Practitioner self-efficacy in identifying, discussing and treating depression in CVD populations may pose a barrier to realisation of routine depression screening and care guidelines in CVD [37]. Notably, health practitioner training has been shown to improve self-efficacy around identifying and managing mental health [39]. Whether concerted efforts to improve self-efficacy of health practitioners may promote mental health information provision and enhance health services, thereby facilitating mental health service use, warrants future examination.

A related barrier was a low awareness of and openness to mental health services. How to best facilitate buy-in to psychology services and enhance mental health uptake remain ongoing challenges for reducing mental health burden globally [40]. Additional strategies to promote mental healthcare in CVD health services appear needed along with ongoing examination of how to better facilitate buy-in. Balancing formal mental health services with peer support, as suggested by the participants here and implemented in some instances (e.g., the Italian best practice guidelines [13]) may provide a means of better engaging and supporting patients. Low openness to mental healthcare may partly stem from the lack of information provision regarding mental health and services. Increased information regarding expectations around mental healthcare from referrers may also assist patients in considering access; yet may remain limited by referrer lack of knowledge or confidence without dedicated practitioner training [39]. Improved communication from referrers, including a steady stream of communication, has previously been suggested as a means to improve mental health services [41] and may be a means of supporting CVD patients in accessing services. Fragmented service delivery can also result in low uptake and understanding of mental health services [40]. Better integrated healthcare, such as through collaborative care might be best placed to overcome this, along with other barriers to uptake, by providing comprehensive and integrated care for both physical and psychological disorders [42]. Collaborative care has been effectively implemented in some settings, and is recommended by the 2019 Lancet Psychiatry Commission for managing comorbid physical health conditions [43]. Further implementation of collaborative care across healthcare systems may be beneficial. Collaborative care can also facilitate the upskilling of staff regarding mental health [44], which may help to redress information provision barriers. The practical barriers identified in thus study, such as high volume of medical appointments associated with physical health conditions, along with transport and access issues, may also be partly overcome with a blended collaborative care approach [42].

Participants reported that the timing of mental health screening and provision of information and referral was a barrier for seeking mental healthcare. Repeatedly, interviewees indicated that screening and referral had taken place too soon, with interviewees reporting that their mental health often worsened over time post-CVD onset. Currently, many international CVD guidelines recommend routine depression screening, however, there is inconsistency in the timing of such initiatives ranging from the index CVD admission and the short-term [8, 10, 13] to unspecified timeframes [9, 11]. The early stages of CVD or admission screening was considered problematic by the patients, which might partly explain the low conversion of positive depression screen to service uptake repeatedly observed [15, 16]. Later screening and/or longer term mental health follow up to monitor mental health needs of the CVD population beyond the index admission or rehabilitation setting [45] may aid in meeting mental health needs. Currently, there is sparse data on repeated and routine screening for mental health in CVD populations. One-half to two-thirds of common mental health disorders are undiagnosed by general practitioners [46–48] and thus later presentations of mental health concerns may not be detected during follow-up in primary care for CVD patients. Conversion of referral to mental health service use for later screen and information provision would be valuably explored in future studies.

Informal support such as through support networks was considered preferable to formal mental health services by most interviewees. Peer support networks have been shown to improve mental health and quality of life, especially where roles and relationships are well defined [49]. Within CVD populations, some peer support networks have been established. For example, a peer support network has been shown to improve psychological wellbeing among adults with congenital heart disease in Italy [50], where guidelines recommend the role of peer networks [13]. Similarly, a preference for mental and physical health interventions with a social support element has also been expressed by primary care CHD patients [22]. Further, peer support networks may aid in normalising psychological distress and enhancing information provision and mental health awareness, which may reduce barriers to accessing mental healthcare such as stigma, fear of disclosure and concerns around judgement from others [24]. Expanding and developing peer support networks may also be beneficial by allowing group members to meet others who have sought mental health care, given that knowing someone who has sought help is related to increased positive expectations and attitudes to mental health services and intentions to seek help [51]. However, to date, outcomes, effectiveness and beneficial components of peer support networks remain less well researched [52] and ongoing implementation and evaluation of peer support networks for CVD patients may be beneficial. Future studies assessing effectiveness, feasibility and uptake of peer support networks including comparisons with formal mental health services would be valuable.

Only two participants in this study identified themselves as having a mental health condition, further limiting help seeking intentions. The limited self-identification with diagnostic terms might partly be due to stigma, with self-stigma regarding mental health associated with reduced help seeking behaviour [53]. Further, CHD patients understood depression in broader social terms [22], and thus the CVD sample interviewed might also not identify with psychiatric nomenclature for understanding their own experiences. Relatedly, stoicism was commonly observed in the interviews, with patients expressing a belief that they should be able to cope over time. This finding echoes prior research showing that CVD patients are reluctant to disclose symptoms of their mental health because they hope, that as time passes, the worries will dissipate [54]. Stoicism is consistent with the broader barriers to help seeking literature [23] and impairs help seeking behaviours [24]. Low awareness of mental health disorders and limited mental health literacy may also underlie the lack of self-identification with, and help seeking for, mental health disorders, as has been found in other populations [53, 55]. Therefore, in order to better engage CVD patients, a breadth of inclusive terminology, such as ‘learning to cope’ and ‘difficulty adjusting’ may better resonate with and engage the broader CVD population and assist in uptake of mental health messages. Engaging in patient terminology around mental health further extends the use of patient-centred communication as a means to promote satisfaction and service utilisation in cardiac settings [10]. Further research exploring outcomes and acceptability of broader terms for engaging patients in mental health support would be useful and may then be incorporated into recommended questions for assessing psychosocial risk amongst CVD patients (e.g., [56]). Concomitantly, diagnostic nomenclature might be better normalised should patients have access to CVD-peer supports, as was their preference here; and the benefit of such terminology for stigma reduction in CVD patients would also be usefully explored.

### Strengths and limitations

The present study provides an insight into a range of common help seeking barriers reported by a sample of CVD patients with clinically significant psychological distress. However, it must be acknowledged that this sample is self-selecting based on those who responded to recruitment materials about impacts of CVDs on wellbeing and quality of life, and thus may not reflect the experience of all adults with CVDs and poor mental health. However, the sample was reflective of mental health outcomes of the CVD population and provided insight into the experiences of those who had not sought help despite referral and elevated psychological distress symptoms. Similarly, the sample represented a mix of CVDs and clinical presentations, thus participants are likely to have had varied experience of their CVD and related impacts on wellbeing. This study valuably presents patterns of experience that were observed across a range of CVDs and thus insights may be able to inform health service provision across a range of CVDs. Given that males and older adults are less likely to seek help for mental health symptoms [24], a strength of the study was that the sample also represented a range of ages and mix of genders. Thus the sample represents a variety of experiences, supporting the naturalistic generalisability and utility of these findings beyond the interviewed sample (for a full discussion in generalisability of qualitative designs, see [57]). A further limitation is that participants are from one country and thus reflect one healthcare system, additional or alternative barriers may present in different cultures and health contexts. However, the present findings have potential implications across a range of healthcare settings, with the gap between positive screen and uptake of mental health care seen across multiple healthcare systems [15–18, 20].

## Conclusions

Mental health disorders such as depression and anxiety are commonly comorbid with CVDs and portend increased risk for morbidity and mortality. Despite guidelines recommending routine depression screening in many countries, uptake of mental healthcare referrals remains low in CVD populations. The current study has identified a range of barriers that may inhibit CVD patients from seeking help for their mental health, that are specific to this population and extend beyond barriers to mental health help seeking already identified in the general population. Findings suggest the timing of screening and referral did not facilitate accessing support. Thus longer term follow-up and later screening may be better placed to reduce mental health burden in this population. Additionally, a preference for peer support over traditional mental healthcare was reported. A low level of mental health literacy was evident and this further compounded reluctance to seek help, indicating a greater need for information provision in healthcare settings. A diversity of language beyond diagnostic categories may also be better suited to engage patients.

## Supplementary Information

**Additional file 1.**

## Data Availability

The datasets generated and/or analysed during the current study are not publicly available to ensure participant privacy, but are available from the corresponding author on reasonable request.

## Abbreviations

CVD: Cardiovascular diseases
CHD: Coronary heart disease
AF: Atrial fibrillation
CABG: Coronary artery bypass graft
MI: Myocardial infarction
PCI: Percutaneous coronary intervention
SCA: Sudden cardiac arrest
