# Supplementary material for: Screening and referral is not enough: a qualitative exploration of barriers to access and uptake of mental health services in patients with cardiovascular diseases
Source: BMC Health Serv Res. 2021 Jan 8;21:49. doi: 10.1186/s12913-020-06030-7 (PMC7796597; doi:10.1186/s12913-020-06030-7)
Supplement: Supplementary file 1 — Additional file 1. [file 12913_2020_6030_MOESM1_ESM.docx]

**Appendix A**

**Outline of Interview Questions**

1. Can you tell me what it is like living with cardiovascular disease?
2. How has living with CVD affected your lifestyle?
3. Can you describe how you felt when you found out that you had CVD?
4. Did you speak to anyone including your doctors about your thoughts/feelings/concerns at the time or later? Can you tell me your experience with this?
5. What factors encouraged you to seek / not seek help from others?
6. Did your doctor or specialist ever assess or offer to assess you for depression or anxiety or another mental health issue in relation to your CVD?
   1. How long after your diagnosis, did you receive a mental health assessment?
   2. Can you tell me about this this?
7. Have you ever been referred by your medical practitioner or heart specialist to see a counsellor, social worker or psychologist?
   1. How long after your diagnosis of CVD did this occur?
8. Did you follow up on the referral?
   1. What encouraged you to follow up on the referral/what stopped you from following up on the referral?
   2. Can you tell more about this?
9. How has attendance to psychological services affected your quality of life living with CVD?
10. Did you ever seek mental health services for yourself regarding your CVD?
    1. What encouraged you to seek help and can you tell me about this?
    2. What stopped you from seeking help?
    3. How often do you see a counsellor or psychologist?
11. What would have made it easier for you to consider seeing a psychologist?
12. What changes / support would you like to see in regard to the treatment and care of people living with CVD who are experiencing mental health concerns?
13. Would you recommend counselling for other people struggling with anxiety and depression who have received a diagnosis of CVD. Why? Why not?
14. Do you think it is important for doctors to enquire about the mental health of their patients after they have received a diagnosis of CVD? Why? Why not?
15. In what ways could health services improve the quality of life for people living with CVD and mental health concerns?
